# Sustainable Sugar Syrup Production from Rice Husk Using *Aspergillus niger* Carbohydrase

**DOI:** 10.1021/acsomega.5c03929

**Published:** 2025-07-16

**Authors:** Fabiane N. Silva, Igor C. F. Sampaio, João Marcos de S. Chaves, Luise de O. Sena, Iana T. Emmerich, Gabriel Lucas S. de Jesus, Floriatan S. Costa, Adriano A. Mendes, Jailma B. dos Santos, Isabela V. L. de Moura, Iasnaia Maria de C. Tavares, Muhammad Irfan, Marcelo Franco

**Affiliations:** a Department of Exact Sciences and Natural, State University of Southwest Bahia, Itapetinga 45700-000, Brazil; b Biotransformation and Organic Biocatalysis Research Group, Department of Exact Sciences, Santa Cruz State University, Ilhéus 45654-370, Brazil; c Department of Biological Sciences, 74361 Santa Cruz State University, Ilhéus 45654-370, Brazil; d Institute of Health Sciences, Federal University of Bahia, Salvador, Bahia 40110-100, Brazil; e Departament of Chemistry, Federal University of Paraná, Curitiba 80060-000, Brazil; f Institute of Chemistry, 74347 Federal University of Alfenas, Alfenas 37130-001, Brazil; g Institute of Chemistry and Biotechnology, 28112 Federal University of Alagoas, Maceió 57072-900, Brazil; h Department of Human Sciences and Technology, Campus XXI, University of Bahia State, Ipiaú, BA 45570-000, Brazil; i Departament of Biotechnology, Faculty of Science, University of Sargodha, Sargodha 40100, Pakistan

## Abstract

Cellulases play a crucial role in cellulose degradation and lignocellulosic biomass conversion. The cost-effective production of these enzymes can be achieved using *Aspergillus niger* in solid-state fermentation with low-cost substrates. In this study, the optimal composition of the substrate mixture (80% cocoa bean shell and 20% forage palm) was determined by using the Simplex-Centroid design. The optimal fermentation conditions (28 °C, 60% moisture for 96 h) were established through the Doehlert design. Enzymatic characterization revealed activity over a broad pH range (5–9) for up to 5 h, a thermal stability of 70% at 70 °C, and halophilic properties, with a 278% increase in activity in the presence of NaCl. Additionally, the enzymes were activated by Ca^2^^+^ (+60%) and DMSO (+69%). The carbohydrase blend (CB) effectively hydrolyzed rice husk, generating 13 mg of reducing sugars per gram of biomass. HPLC analysis identified glucose (7.24 mg/L) and fructose (11.21 mg/L), confirming the synergistic activity of the endoglucanase and isomerase enzymes. These results underscore the innovative and biotechnologically relevant nature of the enzymatic formulation, particularly in the sustainable production of fructose-rich syrup from rice husk. The approach holds significant promise for applications in bioenergy generation and the valorization of food waste.

## Graphical abstract

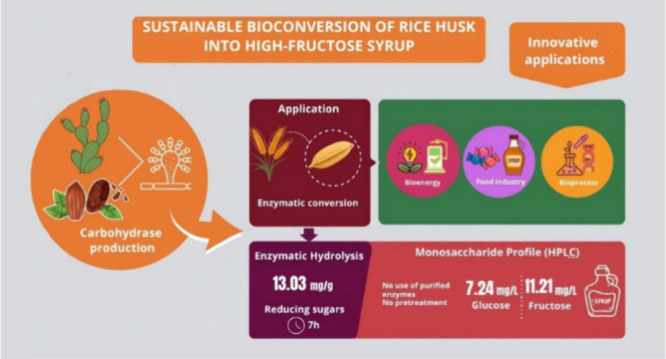

## 1 Introduction

Cellulases constitute a complex of enzymes essential for converting cellulose into glucose, thereby enabling numerous applications in various raw materials containing this polymer, such as the fermentation of its monomers for second-generation bioethanol production.^1^ Among these enzymes, endoglucanases (EC 3.2.1.4) play a pivotal role by hydrolyzing random internal β-1,4-glycosidic bonds within cellulose molecules.^2^

As the third largest group of enzymes used in the industrial sector,^3^ cellulases play a fundamental role in biofuel production, animal feed, and the textile industry. The cellulase market, valued at 1.68 billion dollars in 2020, is projected to experience robust growth, reaching 2.45 billion dollars by the end of 2026, driven by a compound annual growth rate of 5.5% between 2021 and 2026.^4^ However, large-scale cellulase production still faces challenges due to high production costs, the need for stable enzymatic formulations, and variability in the raw material composition. Developing economically viable processes that utilize low-cost food waste as substrates is, therefore, a crucial step in expanding their industrial applications.^1^

Microorganisms exhibit advantageous characteristics for enzyme production due to their product stability and cost-efficient cultivation, facilitating the optimization of industrial processes.^5^ Fungi, bacteria, actinomycetes, and yeasts stand out as prominent cellulase producers, paving the way for a commercially viable and scalable system.^4^ Among these, genera *Trichoderma* and *Aspergillus* are the most commonly utilized.

Solid-state fermentation (SSF) emerges as an efficient and economical method for enzyme production.^6^ SSF enables the production of endoglucanase from various microorganisms and raw materials, such as *Aspergillus oryzae* ATCC 10124 and *Penicillium roqueforti* ATCC 10110 cultivated on rice husk and cocoa husk.^7^ These examples highlight the potential of SSF-based technologies to utilize promising lignocellulosic raw materials in bioprocesses, such as cocoa bean shell (CBS), rice husk (RH), and forage palm (FP).

However, the heterogeneity and variable composition of these food waste pose challenges to maintaining consistent enzymatic yields, reinforcing the need for process optimization strategies.^7^

Optimization of process parameters is crucial for enhancing production and reducing the enzymatic production costs. Experimental design provides greater efficiency compared to the One Factor at a Time (OFAT) approach, particularly in evaluating the impact of multiple factors on a dependent variable while conserving resources such as the number of experiments, time, and materials. Response surface methodology (RSM) is a systematic technique widely applied in processes such as enzyme production to optimize multiple factors and investigate their interactions.^8^

The Simplex-Centroid mixture design, a statistical technique within RSM, aims to refine raw material combinations to determine the ideal composition.^6^ This approach has been employed to optimize the blend of food waste, such as sugar cane bagasse, cocoa husk, and peanut shell, to maximize xylanase production by *Aspergillus oryzae* under SSF conditions and to assess the interaction effects within the mixture components.^9^ However, studies focused on optimizing endoglucanase production using complex mixtures of food waste remain limited.

The Doehlert design, another statistical approach, optimizes process variables such as time, temperature, and humidity. It functions as a second-order experimental design capable of evaluating multiple variables simultaneously. Utilizing the Doehlert design can result in more efficient and cost-effective processes, as highlighted by Nogueira et al.^10^ Marques et al.^11^ applied this design to analyze temperature and incubation time parameters for xylanase and endoglucanase production by *Penicillium roqueforti* ATCC 10110 under SSF conditions.

In the context of processing and utilizing agro-industrial waste, the filamentous fungus *Aspergillus niger* (*A. niger*) stands out as a versatile biofactory for producing essential enzymes such as endoglucanase.^12^ Its significant industrial relevance has driven efforts to enhance enzyme production methods for improved cost efficiency and better utilization of food waste. *A. niger* has been employed to produce enzymes from various raw materials, including cactus pear,^13^ prickly palm cactus,^12^ and mango residues,^13^ targeting applications such as essential oil extraction^14^ and saccharification processes.^15^

Despite advancements in enzymatic production strategies, a significant gap remains in optimizing endoglucanase production from *A. niger* using complex mixtures of lignocellulosic biomass. Given the broad spectrum of applications and the industrial significance of endoglucanase, it is essential to identify lignocellulosic raw material combinations that effectively facilitate its production, thereby enhancing its utilization. Although these materials are widely available, their potential for large-scale enzyme production remains underexplored.

In this context, this study aimed to produce and optimize a crude enzymatic extract, obtained by solid-state fermentation with *A. niger* ATCC 1004, using a low-cost lignocellulosic biomass mixture of forage palm (FP), cocoa bean shell (CBS), and rice husk (RH). Endoglucanase activity was used as the response variable for the optimization of the fermentative process, also being quantified and biochemically characterized in the extract, with evaluation of its thermal stability, pH variation, and tolerance to salts and organic solvents. Finally, the crude enzymatic extract was applied in the direct saccharification of RH, focusing on the production of a high-value sugar syrup, pointing to an innovative and sustainable approach in the valorization of lignocellulosic biomass.

## 2 Material and Methods

### 2.1 Lignocellulosic Biomass

Rice husk (RH) was acquired from a rice processing agro-industry situated in the western region of Bahia, Brazil. Cocoa bean shell (CBS) was sourced from the Barry Callebaut Company located in Itabuna, Bahia, Brazil. Forage palm (FP) was generously provided by the State University of Southwest Bahia (UESB) in Itapetinga, Bahia, Brazil.

Initially, these materials were washed with distilled water and dried in a forced air oven (model 420-6D, Nova Ética, Vargem Grande Paulista, São Paulo, Brazil) at 50 °C for 72 h. Subsequently, they were ground in a Willey knife mill (model Willey Mill, ACB Labor, Brazil) to obtain a particle size of approximately 2 mm. The resulting material was stored in a sealed plastic container in a dry place. The moisture content of the biomass was assessed using an infrared moisture analyzer (ID200, MARTE, São Paulo, Brazil). Bromatological analyses were conducted to determine the lignin, cellulose, hemicellulose, dry matter (DM), mineral matter (MM), crude protein (CP), neutral detergent fiber (NDF), acid detergent fiber (ADF), and ether extract (EE) contents.^16^ The results were expressed as a percentage (g/100 g) of dry matter.

### 2.2 Solid-State Fermentation

The solid-state fermentation process was carried out in 125 mL Erlenmeyer flasks containing 5 g of a biomass mixture. Inoculation was performed with 10^7^ spores of *A. niger* ATCC 1004 per gram of biomass. The tests were conducted over a period of 144 h to determine the optimal time for the maximum endoglucanase activity. Samples were collected every 24 h to measure enzyme activity while maintaining a constant temperature (20 °C) and humidity (70%). After fermentation, distilled water was added to the fermented raw material, and the mixture was agitated at 30 °C and 200 rpm for 30 min. The aqueous phase was then separated by mechanical pressing and subjected to centrifugation.^17^ The supernatant was collected and used as the crude enzymatic extract in the saccharification assays

#### 2.2.1 Multivariate Optimization

The optimization of endoglucanase production followed a two-stage process. First, a Simplex-Centroid design was employed to optimize the blend of lignocellulosic materials with four levels for each factor (CBS, FP, RH). This approach aimed to identify the ideal mixture composition for enzyme production, with nine runs in the experimental matrix (Table 1).

**Table 1:** Experimental Matrix of Simplex-Centroid Mixture Design and Experimental Response Presented in Real and Encoded Values

experiment number	cocoa bean shell—A (g)	forage palm—B (g)	rice husk—C (g)	endoglucanase (U/mL)
1	5 (1)	0 (0)	0 (0)	0.77 (±0.01)
2	0 (0)	5 (1)	0 (0)	0.60 (±0.00)
3	0 (0)	0 (0)	5 (1)	0.00 (±0.00)
4	2.5 (0.5)	2.5 (0.5)	0 (0)	0.57 (±0.02)
5	2.5 (0.5)	0 (0)	2.5 (0.5)	0.47 (±0.02)
6	0 (0)	2.5 (0.5)	2.5 (0.5)	0.31 (±0.01)
7*	1.66 (0.33)	1.66 (0.33)	1.66 (0.33)	0.26 (±0.01)
8*	1.66 (0.33)	1.66 (0.33)	1.66 (0.33)	0.25 (±0.03)
9*	1.66 (0.33)	1.66 (0.33)	1.66 (0.33)	0.23 (±0.00)

In the second stage, the Doehlert design was used to optimize the fermentative process variables, specifically temperature and humidity, also with nine runs in the experimental matrix. Endoglucanase activity was adopted as the response variable. Optimal conditions were determined based on the model generated from the Doehlert design (Table 2). Regression models were adjusted to account for significant interaction effects among variables, and the quality of fit was evaluated using the coefficient of determination (*R* ^2^) and analysis of variance (ANOVA). Statistical processing and data analysis were performed by using Statistica 12 software.

**Table 2:** Experimental Doehlert Matrix, Featuring Both Real and Encoded Values, Was Utilized to Optimize the Experimental Parameters for Solid-State Fermentation Aimed at Producing Endoglucanase from *A. niger* ATCC 1004

experiment number	temperature—A (°C)	humidity—B (%)	endoglucanase (U/mL)
1	36 (1)	70 (0)	0.32 (±0.00)
2	32 (0.5)	80 (0.866)	0.34 (±0.01)
3	20 (−1)	70 (0)	0.27 (±0.00)
4	24 (−0.5)	60 (−0.866)	0.42 (±0.03)
5	32 (0.5)	60 (−0.866)	0.40 (±0.02)
6	24 (−0.5)	80 (0.866)	0.30 (±0.01)
7*	28 (0)	70 (0)	0.40 (±0.01)
8*	28 (0)	70 (0)	0.41 (±0.00)
9*	28 (0)	70 (0)	0.38 (±0.01)

#### 2.2.2 Determination of Endoglucanase Activity

Endoglucanase activity was assessed by measuring the reducing sugars released from the hydrolysis of 2% (w/v) carboxymethyl cellulose (CMC) (Cromoline, Diadema, Brazil) diluted in 0.1 mol/L sodium acetate buffer at pH 5.0, using the dinitrosalicylic acid (DNS) method (Sigma-Aldrich, St. Louis, USA).^18^

### 2.3 Biochemical Characterization

#### 2.3.1 Effect of Temperature and Thermal Stability with Estimation of Thermodynamic Parameters

Endoglucanase activity was assessed at temperatures ranging from 40 to 80 °C for 15 min.^7^ Thermal stability was evaluated at 60, 70, and 80 °C in sodium acetate buffer at pH 5.0 (ionic strength 0.1 mol/L) at 1 h intervals. Residual enzymatic activity in CMC hydrolysis at the temperature of the maximum activity was considered to be 100%, and the results were expressed as relative activity (%).

For thermal inactivation analysis, the model that exhibited the strongest correlation with the data, as determined by the correlation coefficient (*R* ^2^), was used to derive kinetic (thermal inactivation constant, *K*_d_) and thermodynamic parameters (activation energy, enthalpy, Gibbs free energy, and entropy). The *K*_d_ value was calculated using the nonlinear exponential decay model proposed by Sadana and Henley^20^ and the linear decay model^19^ as presented in eqs 1 and 2, respectively. Data analysis was performed using Origin Pro software, version 8.0.

$$a_{r}=\propto +\left(1+\propto \right)xe^{-k_{d}xt}$$ 1

$$A=A_{0}e^{\left(-kdt\right)}$$ 2

where *a*_n_ is the relative activity (dimensionless); α is the ratio of the specific activity of the final state (*E*_1_) to the initial state (*E*); *K*_d_ is the inactivation constant (min^–^^1^); *t* is the thermal inactivation time (min); and *K*_d_, *T*, *K*_b_, *h*, Δ*S* ^#^, Δ*H* ^#^, and *R* represent the specific reaction rate, absolute temperature, Boltzmann’s constant, Planck’s constant, inactivation entropy, inactivation enthalpy, and gas constant, respectively.

The thermal inactivation energy (*E*_d_) of endoglucanase was determined by plotting the natural logarithm of the thermal inactivation constant (ln *K*_d_) against the inverse of the absolute temperature (1/*T*), as indicated in eq 3.^21^

$$\ln\;E_{d}=\ln\;A-\frac{E_{d}}{R}\frac{1}{T}$$ 3

where *A* is the frequency factor (s^–1^).

The enthalpy values (Δ*H* ^#^—kJ/mol), Gibbs energy (Δ*G* ^#^—kJ/mol), and entropy (Δ*S* ^#^—kJ/mol.K) for the thermal inactivation of the enzyme were determined using the corresponding equations eqs 4, 5, and 6, respectively.^21^

$$\Delta H^{\#}=E_{d}-RT$$ 4

$$\Delta G^{\#}=-RT\ln\left(\frac{k_{d}h}{k_{B}T}\right)$$ 5

$$\Delta S^{\#}=\Delta H^{\#}-\Delta G\frac{\#}{T}$$ 6

where *T* is the absolute temperature (K), *h* is Planck’s constant (11.04 × 10^–36^ J/s), and *k*_B_ is Boltzmann’s constant (1.38 × 10^–23^ J/K).

#### 2.3.2 Stability and pH Effect

The optimal pH for the maximum CB activity was investigated using buffers covering pHs 3–9:^7^ sodium citrate buffer (0.05 mol/L; pHs 3, 4, and 5), sodium phosphate buffer (0.05 mol/L, pHs 6 and 7), and Tris-HCl buffer (0.05 mol/L, pHs 8 and 9). The reaction was conducted at 50 °C for 5 h, with sampling at 1 h intervals. Results were expressed as relative activity (%) with the highest enzymatic activity set as 100%.

#### 2.3.3 Effect of Salts and Organic Compounds

The impact of various salts and organic compounds on the endoglucanase activity was assessed. The tested additives included iron sulfate (FeSO_4_), cobalt sulfate (CoSO_4_), calcium chloride (CaCl_2_), magnesium sulfate (MgSO_4_), aluminum nitrate [Al­(NO_3_)_3_], sodium sulfate (Na_2_SO_4_), magnesium chloride (MgCl_2_), lead acetate [Pb­(C_2_H_3_O_2_)_2_], calcium carbonate (CaCO_3_), copper sulfate (CuSO_4_), zinc acetate [Zn­(C_2_H_3_O_2_)_2_], sodium carbonate (Na_2_CO_3_), zinc sulfate (ZnSO_4_), iron chloride (FeCl_2_), potassium chloride (KCl), EDTA (ethylenediaminetetraacetic acid), Triton X-100, and Trolox (6-hydroxy-2,5,7,8-tetramethylchroman-2-carboxylic acid).

The experimental setup involved diluting the enzymatic blend in a 2 mmol/L solution of each additive in sodium acetate buffer at pH 5 (ionic strength = 0.1 mol/L), followed by enzymatic activity measurement. Results were expressed as relative activity (%) compared to the activity of endoglucanase without additives, which was considered 100%.^6^

#### 2.3.4 Solvent Effect

The effect of organic solvents on endoglucanase activity was evaluated using dimethyl sulfoxide (DMSO), dimethylformamide (DMF), methanol, acetonitrile, acetone, ethyl ether, dichloromethane, and hexane.^6^ Each solvent was added to 1 mL of 0.1 mol/L sodium acetate buffer (pH 5) to achieve final concentrations of 20 and 30% (v/v). All samples were subjected to agitation before the endoglucanase activity was measured. Results were expressed as relative activity (%) with the enzymatic activity of the sample without organic solvents serving as the control (100%).

#### 2.3.5 Effect of NaCl Addition

The salt resistance of *A. niger* ATCC 1004 endoglucanase was evaluated by measuring its hydrolytic activity at varying NaCl concentrations.^6^ Distilled water was used as the solvent to test NaCl concentrations of 0, 0.01, 0.05, 1, 2, 3, 4, 5, and 6 mol/L under optimized assay conditions following the standard enzyme quantification method.

Results were expressed as relative activity (%) with 100% representing the enzymatic activity in the absence of NaCl (0 mol/L).

### 2.4 Enzymatic Saccharification

The enzymatic blend derived from *A. niger* ATCC 1004 was used for RH saccharification. The reaction mixture contained 60 mL of the enzymatic extract in a sodium citrate buffer (0.05 mol/L, pH 5). Control 1 consisted solely of the enzymatic blend, without RH, while control 2 contained only RH and 60 mL of buffer.

Saccharification was carried out in a water bath with agitation at 50 °C for 7 h, with aliquots collected at 1 h intervals. After the reaction, the supernatant was used to quantify the released reducing sugars based on a standard glucose curve (1 mmol/mL). The sugar content was analyzed via HPLC using refractive index (RI) detection.

The chromatographic system included a ProStar 210 pump (Varian, Palo Alto, USA), a manual injector 80765 (Hamilton, Indaiatuba, Brazil) with a 20 μL loop, and an RI detector (model 356 LC). Product identification was performed using an 87H chromatographic column (300 mm length, 7.8 mm inner diameter; MetaCarb, Agilent Technologies, Barueri, Brazil), operating under the following conditions: column temperature set at 55 °C, sulfuric acid (0.005 mol/L) as the mobile phase, and flow rate of 0.70 mL/min. Samples were analyzed both at the initial time point (representing nonsaccharified RH) and after 7 h.^18^ All enzymatic reactions were performed in triplicate.

## 3 Results and Discussion

### 3.1 Lignocellulosic Biomass

The nutritional potential of the raw materials was assessed through physicochemical analyses to determine their suitability as carbon and nitrogen sources for microbial cultivation. The compositions (% by dry weight) obtained from the bromatological analyses of the raw materials are presented in Table 3.

**Table 3:** Composition (% by dry weight) of the Raw Materials Obtained by Bromatological Analysis

	physicochemical composition (%)							
raw materials	NDF	ADF	hemicellulose	cellulose	lignin	CP	EE	MM
FP	23.81 ± 0.02	8.03 ± 0.00	15.84 ± 0.02	7.89 ± 0.00	0.19 ± 0.00	7.27 ± 0.01	0.77 ± 0.01	25.94 ± 0.01
CBS	49.44 ± 0.00	36.69 ± 0.01	11.37 ± 0.01	20.67 ± 0.01	16.67 ± 0.00	21.26 ± 0.00	11.15 ± 0.00	9.91 ± 0.00
RH	91.92 ± 0.00	91.92 ± 0.00	22.07 ± 0.00	22.07 ± 0.00	22.07 ± 0.00	22.07 ± 0.00	0.18 ± 0.02	9.23 ± 0.00

The ratio of these components plays a crucial role in fermentation success.^14^ Due to its structure and lower crystallinity, hemicellulose may serve as the primary carbon source for *A. niger*, followed by cellulose. Proteins in the residues can also be utilized by microorganisms, primarily as a nitrogen source and occasionally as an energy source.

Therefore, achieving a higher ratio of cellulose, hemicellulose, and protein relative to that of lignin is essential for promoting microbial metabolism. Although rice husk contains a higher cellulose content, its elevated lignin levels and lower protein content could hinder the initial microbial activity. Conversely, the low lignin content in forage palm, combined with a balanced ratio of hemicellulose, cellulose, and protein, may provide more accessible carbon and nitrogen sources, reducing the lag phase and enhancing microbial growth.

### 3.2 Solid-State Fermentation

Enzymatic activity assays conducted at various time points revealed that the optimal fermentation duration for the maximum endoglucanase production (0.352 U/mL) was 96 h after which a significant decline in enzymatic activity was observed. This optimal time frame depends on the specific microorganism and cultivation conditions. The *Aspergillus* genus is well-known for its ability to produce endoglucanase from various lignocellulosic raw materials. This capability allows these fungi to degrade polymers such as cellulose, hemicellulose, and lignin present in plant matrices, releasing sugars and other compounds that serve as nutrients for microbial growth.^22^

Previous studies have reported that *A. niger* reaches peak endoglucanase activity at 71.5 h when utilizing mango residues,^13^ while *Botrytis ricini* URM 5627 exhibits the maximum activity at 144 h when cultivated on sugarcane bagasse but shows reduced activity on raw coconut fiber.^17^ Additionally, other studies indicate decreased endoglucanase activity after 141.5 and 192 h when *Aspergillus oryzae* ATCC 10124 was grown on rice husk (RH) and peanut husk, respectively.^7^

The observed enzymatic profile with raw materials, such as prickly pear cactus, mango residues, and sugar cane bagasse, may be attributed to their higher hemicellulose and cellulose content and lower lignin levels. In contrast, compounds such as phenolics, acetyl groups, and lignin can hinder enzymatic activity by acting as physical barriers or inhibitors, potentially leading to cellulase deactivation through adsorption on their surfaces.^23^

On the other hand, the hemicellulose and cellulose content of rice husk RH), peanut husk, and coconut husk exhibited notable differences. During the initial stages of microbial growth, the enzymatic machinery adapts by prioritizing the consumption of the available raw materials. After this adaptation phase (lag phase), metabolic activity and enzymatic secretion increase until they reach their peaks. As the primary carbon source is hydrolyzed and nutrient availability declines, or metabolic byproducts accumulate, potentially inhibiting enzyme production, there is a regulatory decrease in enzyme secretion, leading to a transition toward the production of alternative enzymes.^8,24^

#### 3.2.1 Multivariate Optimization

The strategic selection of mixture composition has been shown to significantly enhance the fermentative process.^9^ Experiments demonstrated that *A. niger* ATCC 1004 can produce endoglucanase using only lignocellulosic raw materials, without the need for additional carbon, nitrogen, or mineral sources. Statistical analysis (Table 4A) confirmed the validity of the mathematical model at a significance level of 0.05, supported by high *R* ^2^ and adjusted *R* ^2^ values (0.999 and 0.997, respectively), indicating a strong correlation with the experimental results.

**Table 4:** Analysis of Variance for the Special Cubic Model for the Centroid Simplex (A) and Analysis of Variance for the Quadratic Model (Doehlert Design) Applied in the Optimization of Experimental Conditions (B)^a^

**source of variation**	**SS**	**Df**	**MS**	calculated *F*	*p*-value	***R*** **^2^**
**A.** **Optimization of raw material composition by centroid simplex**						
regression	0.448	6	0.075	319.8	0.003	0.999
total error	0.001	2	0.001			adjusted *R* ^2^
total	0.449	8	0.056			0.996
**B. Optimization of fermentation conditions by Doehlert design**						
regression	0.023	5	0.005	21.40	0.01	0.973
lack of fit	0.001	1	0.001	0.60	0.52	
pure error	0.001	2	0.001			
total	0.025	8				

a Sum of squares (SS), degrees of freedom (Df), mean square (MS), coefficient of determination (*R* ^2^). *95% confidence level (*p* = 0.05).

The Pareto chart (Figure 1c) identified CBS and RH as key variables for endoglucanase production, whereas FP had a minimal impact. Contour plots revealed that a mixture containing 80% CBS and 20% FP was selected for the study due to its optimal enzymatic activity, with CBS exerting a greater influence on production than that of FP (Figure 1a).

**Figure 1:**
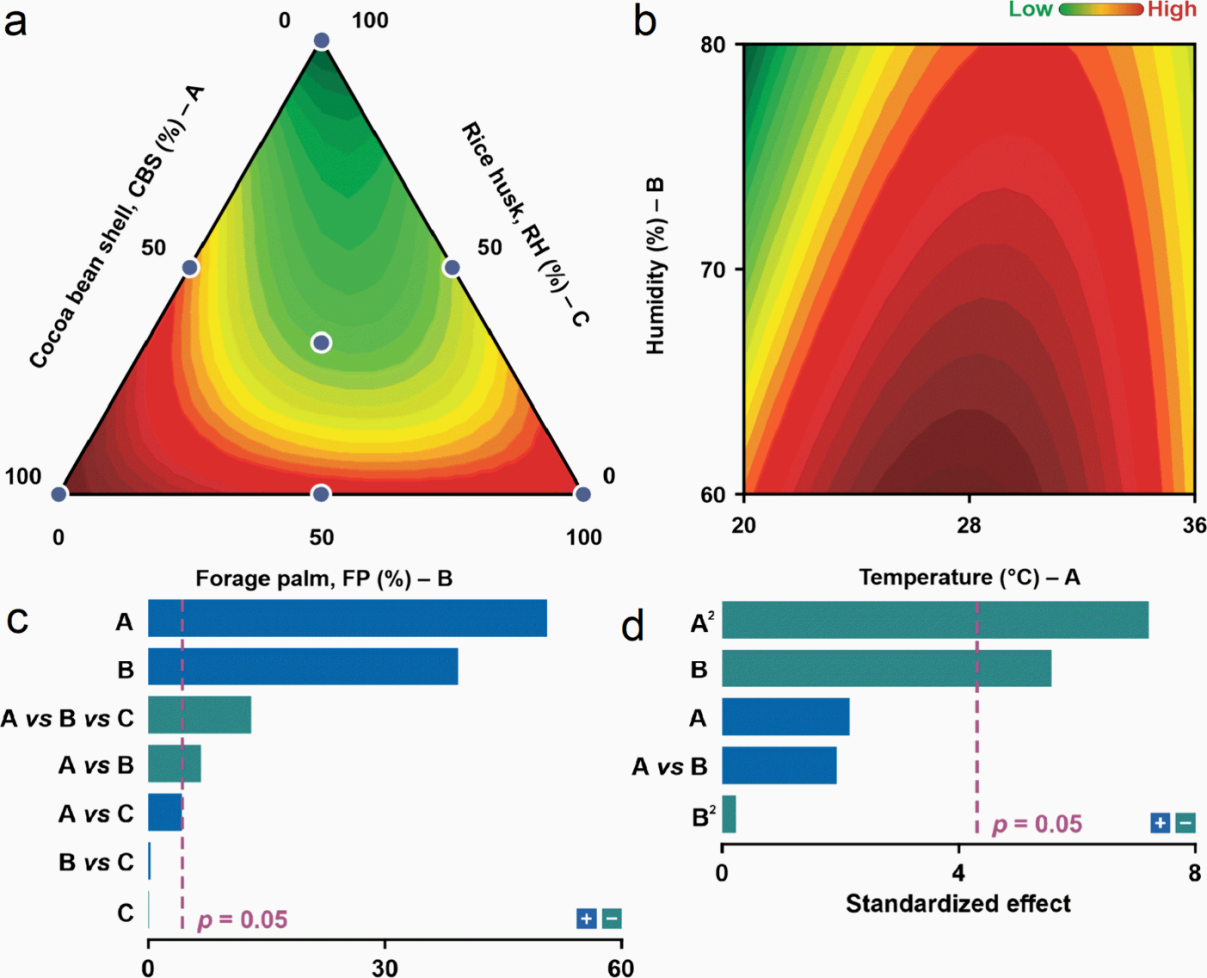
(a) Contour and surface plots obtained from the special cubic model. A—cocoa bean shell; B—forage palm; C—rice husk; (b) contour plot representing interactions between variables with endoglucanase activity as the response, obtained from the quadratic model in Doehlert Design. A—temperature; B—humidity; (c) standardized effects for investigated factors (Pareto plot) in Simplex-Centroid mixture design. A—cocoa bean shell; B—forage palm; C—rice husk; (d) pareto plot obtained from the quadratic model in Doehlert Design. A—temperature; B—humidity.

The experiments conducted following the Doehlert design parameters, using the optimized biomass mixture (80% CBS and 20% FP), revealed consistent secretion of endoglucanase by *A. niger* ATCC 1004 across various combinations of variables. Statistical analysis (Table 4B) confirmed the significance of the mathematical model (*R* ^2^ = 0.973) for endoglucanase production, indicating a strong fit to the experimental data (Figure 1d). Temperature and humidity were found to significantly influence endoglucanase yield, with optimal conditions identified at 60% humidity and 28 °C (Figure 1b). These findings align with previous studies on fungal enzyme production, highlighting the variable nature of optimal conditions influenced by factors such as fungal species and raw material properties.^24^

Temperature and humidity are crucial for fungal growth dynamics and enzyme production, with fluctuations expected to impact enzymatic activities.^11^ Maintaining a balance in selecting optimal conditions is essential to optimize industrial bioprocesses.^25^ Elevated temperatures may trigger heat stress responses in *Aspergillus* specie*s*,^26^ potentially reducing carbohydrase yield due to the diversion of energy and resources toward cytoprotective mechanisms.^27^

### 3.3 Biochemical Characterization

#### 3.3.1 Effect of Temperature and Thermal Stability with Estimation of Thermodynamic Parameters

The investigation into the influence of temperature on endoglucanase activity revealed a notable increase in enzymatic activity with rising temperature, reaching its peak at 50 °C, under which the enzyme showed a maximum activity of 0.450 U/mL. Subsequently, there was a gradual decline in activity as the temperature continued to increase. For instance, *Penicillium roqueforti* ATCC 10110 endoglucanase exhibited its maximum activity at 50 °C,^11^ while *Rhizopus* sp. displayed optimal activity between 50 and 60 °C,^28^ and *Botrytis ricini* URM 5627 also demonstrated peak activity at 50 °C.^17^

Elevated temperatures positively affect catalysis rates in chemical reactions, reducing microbial contamination risk and improving compound solubility, thereby enhancing raw material diffusion in chemical reactions.^24^ Employing optimal temperatures proves effective in enhancing efficiency and reaction rates in catalytic and hydrolytic processes, which is crucial for various chemical reactions in industrial applications.

The enzyme’s optimal temperature is likely influenced by its amino acid sequence and the presence of noncovalently bound carbohydrates.^29^ Enzymes possess specific three-dimensional structures defined by their amino acid sequences, and the stability of these conformations depends on noncovalent interactions such as hydrogen bonds, hydrophobic interactions, and salt bridges. Elevated temperatures can disrupt these interactions, leading to denaturation and consequent loss of enzymatic activity.^29^ The presence of hydrophobic residues and a higher proportion of polar, charged amino acids tend to reduce surface hydrophobicity while increasing the hydrophobicity of the protein core. This contributes to enhanced structural rigidity and thermal stability.^30^

Sequence analysis of the FpCel45 endoglucanase from subfamily C of glycoside hydrolase family 45 (GH45) identified key residues associated with thermostability including Ser18, Thr20, Asn95, Trp98, Cys99, Asn108, His115, and Asp117. Compared with other fungal GH45 enzymes, FpCel45 demonstrated superior resistance to high temperatures. Notably, several thermostable endoglucanases from both mesophilic and thermophilic organisms within the GH45 family have been reported in the literature.^31^ When comparing thermostable to mesostable (nonthermostable) endoglucanases, certain amino acids, particularly methionine (Met) and arginine (Arg), as well as enhanced ionic interactions, appear to play a significant role in increasing thermal stability.^32^

It has been reported that Asp95 and Asp99 are the key amino acid residues responsible for conferring thermostability to endoglucanase from *A. niger*. These residues are situated within the enzyme’s substrate-binding pocket and form salt bridges with Na^+^ and Cl^–^ ions, thereby enhancing both stability and catalytic activity under high-salinity conditions.^31^ While other amino acids may also contribute to the thermostability of this enzyme, their specific roles remain poorly understood. It is hypothesized that they may participate in the formation of hydrogen bonds, hydrophobic interactions, or disulfide bridges, all of which play a role in stabilizing the enzyme’s tertiary structure.^33^

Furthermore, some endoglucanases exhibit a distinctive self-truncation mechanism whereby a portion of the C-terminal region is cleaved. Remarkably, the truncated forms display enhanced specific activity and improved thermostability compared to those of the full-length enzyme. This self truncation occurs spontaneously and is influenced by pH and temperature conditions.^34^

Enzyme inactivation increased with increasing incubation temperatures (Figure 2b–d). The endoglucanase produced in this study demonstrated notable thermal stability at 60 °C, retaining 75% of its activity after 1 h of incubation. At 70 °C, activity declined to 70% over the same period, while at 80 °C, a sharp reduction was observed, with only 20% of the original activity remaining after 30 min. Notably, the thermal inactivation constant (*K*_d_) increased from 0.0301 h^–^^1^ at 60 °C to 0.0381 h^–^^1^ at 80 °C.

**Figure 2:**
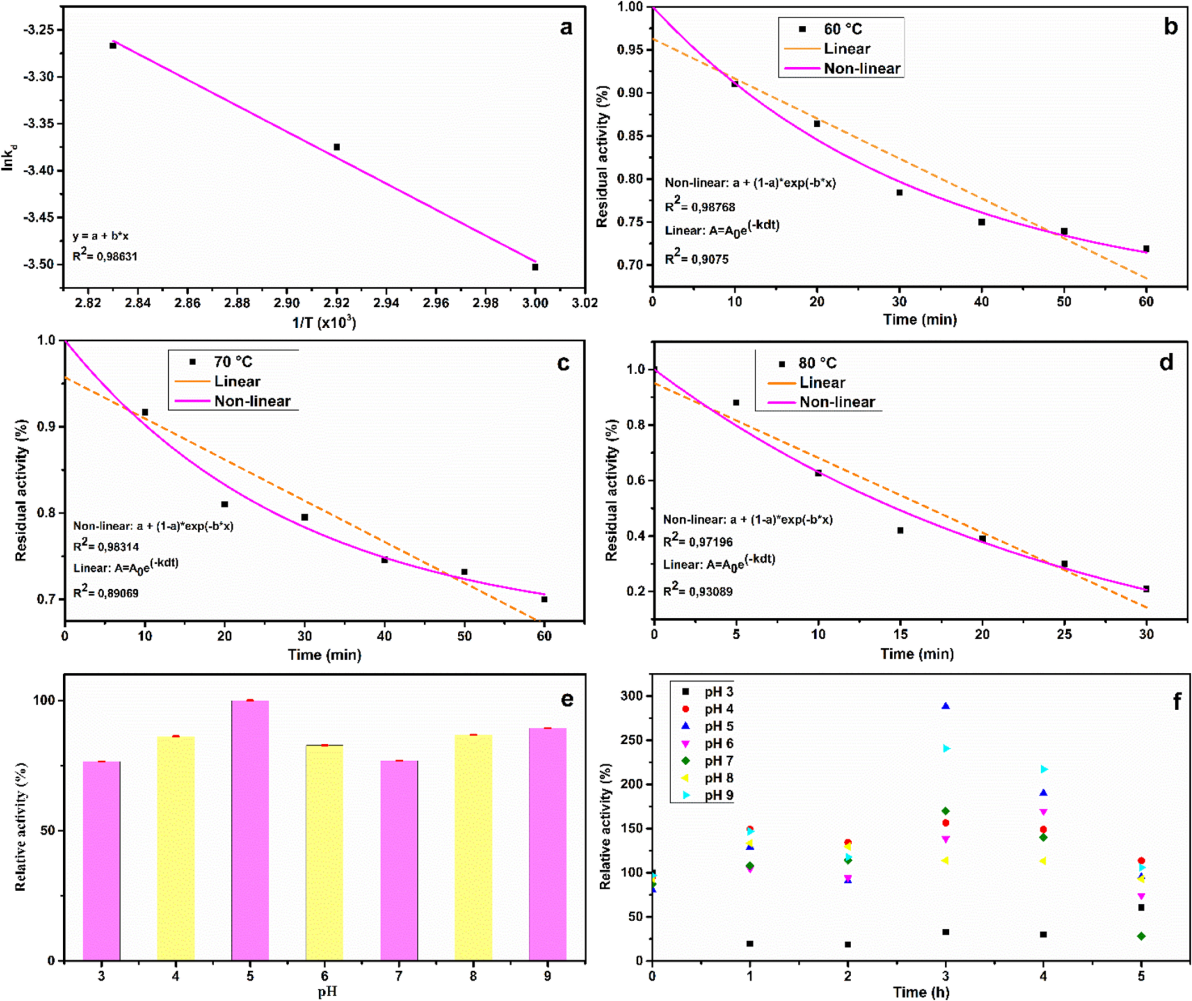
First-order Arrhenius plot depicting the influence of temperature on the activity of crude enzymatic extract from *A. niger* ATCC1004 (a), the activation energy (Ea) for substrate hydrolysis is calculated as the slope multiplied by the gas constant, *R*, where *R* equals 8.314 J/K/mol; thermal inactivation curves of the enzyme blend after incubation at various temperatures (b–d), inactivation tests were conducted by incubating in sodium acetate buffer at pH 5 (ionic strength of 0.05 mol/L); (e) effect of different pH levels on enzymatic activity; (f) stability of the enzymatic extract from *A. niger* ATCC 1004 under varying pH conditions. *Error bars represent standard deviation <0.053.

The inactivation curves exhibited strong correlation coefficients (*R* ^2^ > 0.89; Figure 2a–d). However, the nonlinear exponential decay model yielded a superior fit, as expected due to the complex nature of the enzyme protein structures. Based on these results, the nonlinear exponential decay kinetic model^20^ was selected as the most appropriate for describing the experimental data and was subsequently employed to calculate the enzyme’s thermodynamic inactivation parameters.

Understanding how biomolecules behave under thermal stress requires an analysis of the thermodynamic parameters governing their stability. In this context, the enthalpy (Δ*H* ^#^), entropy (Δ*S* ^#^), and Gibbs free energy (Δ*G* ^#^) of activation for endoglucanase thermal inactivation were estimated from the experimental data (Figure 2a–d), with the calculated values summarized in Table 5.

**Table 5:** Kinetic and Thermodynamic Parameters of the Irreversible Thermal Denaturation of the Crude Endoglucanase from *A. niger* ATCC 1004^a^

temperature					
°C	K	*K*_d_ (min^–1^)	Δ*H* ^#^ (kJ/mol)	Δ*G* ^#^ (kJ/mol)	Δ*S* ^#^ (J/mol/K)
60	333	0.0301	8.74	91.56	–248.7
70	343	0.0342	8.66	94.03	–248.9
80	353	0.0381	8.57	96.53	–249.2

a Legend: the values of *K*_d_ are obtained from Figure 2a. The activation energy for denaturation, Ed, equals the product of the slope and *R* (the universal gas constant), which equals 11.51 kJ/mol.

The endoglucanase produced by *A. niger* ATCC 1004 through solid-state fermentation exhibited an activation energy (Ea) of 11.51 kJ/mol. This value was used to estimate the enthalpy of activation (Δ*H* ^#^) associated with the enzyme’s thermal inactivation process. Given the direct correlation between Ea and Δ*H* ^#^, a higher activation energy reflects a correspondingly higher Δ*H* ^#^. The enzyme demonstrated thermal stability, with Δ*H* ^#^ values of 8.74 and 8.57 kJ/mol at the highest and lowest experimental temperatures, respectively. These positive Δ*H* ^#^ values (Table 5) confirm an endothermic inactivation mechanism, indicating that an energy input is required for enzyme deactivation. The Δ*H* ^#^ also reflects the number of molecular bonds disrupted during inactivation; thus, lower Δ*H* ^#^ values are indicative of greater enzyme resilience to thermal stress.^35^

In contrast, the entropy change (Δ*S* ^#^) decreased with increasing temperature, yielding negative values. A similar pattern was observed for endoglucanase GluCB31 from *Bacillus subtilis*. The role of entropy in thermal inactivation is critical; negative Δ*S* ^#^ values suggest reduced molecular disorder during the transition state, possibly due to protein aggregation that limits the formation of new intra- and intermolecular interactions.^36^

In this study, the Gibbs free energy of activation (Δ*G* ^#^) increased proportionally with temperature, indicating greater resistance to thermal denaturation.^37^ Positive Δ*G* ^#^ values have also been reported for a variety of enzymes. For example, endoglucanase produced by *Aspergillus fumigatus* through solid-state fermentation exhibited Δ*G* ^#^ values of 110, 112, and 107 kJ/mol at 60, 70, and 80 °C, respectively.^29^ A similar upward trend was observed for alkaline protease from *Aspergillus tamarii* URM4634, with Δ*G* ^#^ reaching 96.6 kJ/mol at 80 °C.^38^ Remarkably, glucose isomerase from *Streptomyces roseiscleroticus* exhibited a peak Δ*G* ^#^ value of 24,618.915 kJ/mol at 80 °C.^36^

These findings reinforce that positive Δ*G* ^#^ values are indicative of enzymatic thermal stability at elevated temperatures and that the inactivation process is nonspontaneous. Moreover, the enzyme’s resistance to thermal unfolding suggests an inherent ability to withstand the significant energy input required for denaturation, thereby characterizing it as thermostable.^39^

Thermostable cellulases are recognized for their industrial robustness and versatility, offering significant advantages over conventional enzymes. Their high thermal tolerance, resistance to denaturing agents, prolonged catalytic activity, and reduced contamination risks render them particularly suitable for diverse industrial applications. In the saccharification of lignocellulosic biomass, these enzymes provide enhanced stability, elevated specific activity, and greater operational flexibility. This translates into lower enzyme loading, shorter hydrolysis times, and superior performance under high-temperature conditions. Owing to their increased efficiency and associated economic benefits, thermostable cellulases are positioned to significantly advance the biofuels industry and the broader development of products derived from lignocellulosic biomass.^30^

#### 3.3.2 Stability and pH Effect

The optimal pH for the maximum endoglucanase activity from *A. niger* ATCC 1004 was determined to be pH 5, which was defined as 100% for relative activity comparisons (Figure 2e). Although similar activity levels were observed at pH 9, the enzyme exhibited a superior performance under acidic conditions. Notably, enzymatic activity declined by no more than 20% across the other tested pH ranges, demonstrating broad pH tolerance (Figure 2e). This behavior is consistent with the acidophilic nature commonly associated with fungal endoglucanases, as previously reported for *Aspergillus oryzae* ATCC 10124 ^7^ and *Penicillium roqueforti* ATCC 10110.^11^

A similar trend was observed in the pH stability assay conducted over a 5 h period (Figure 2f). During initial incubation in carboxymethylcellulose (CMC), the endoglucanase exhibited a lag phase with low initial activity (time 0 h, Figure 2f), followed by an increase in catalytic performance. This behavior may be attributed to reversible binding of the enzyme to the substrate, inducing conformational changes that enhance catalytic efficiency over time.^40^ A comparable profile was reported for the endoglucanase from *Aspergillus oryzae*, in which enzymatic activity initially declined before reaching peak levels in the CMC medium.^41^

Following the first hour of incubation, a substantial increase in relative activity was observed across all pH ranges, with the exception of pH 3, which exhibited a sharp decline of 80% (Figure 2f). In contrast, all other pH conditions showed relative activities exceeding 100%. Between the 3rd and 4th hours of incubation, assays conducted at pHs 4, 5, 7, and 9 demonstrated increases in relative activity surpassing 50%, reinforcing the positive influence of varying pH conditions. Notably, pHs 5 and 9 recorded the highest relative activities during the experiment, reaching 288 and 240% at 3 h, respectively.

A marked decrease in activity was detected in the final hour of incubation (5 h). Nevertheless, stability tests at pHs 9 and 4 retained relative activities above 100% until the end of the assay, while pH 5 maintained a relative activity of 95%. The endoglucanase complex produced by *A. niger* ATCC 1004 exhibited robust activity across a broad pH spectrum, both acidic and alkaline, suggesting the presence of multiple isoenzymes or versatile catalytic subunits with distinct pH optima.

This acidophilic/alkaliphilic behavior offers significant advantages in industrial applications, particularly in processes involving the enzymatic hydrolysis of lignocellulosic biomass. Such versatility is especially beneficial in contexts where pretreatments with acids or ionic liquids are employed to liberate fermentable sugars. Enzymes that remain active under extreme pH conditions eliminate the need for pH adjustment postpretreatment, thereby reducing operational costs and streamlining process efficiency.^42^

When compared with previously studied enzymes, the enzymatic blend investigated here demonstrated superior stability. *Penicillium roqueforti* ATCC 10110 exhibited stability at pHs 4 and 8, retaining approximately 80% of its enzymatic activity after 5 h of incubation at 50 °C.^43^ A single study also evaluated endoglucanases from *A. niger* and *Rhizopus sp.*, reporting that the *A. niger* enzyme remained stable for 4 h with nearly 100% relative activity at pHs 5 and 6, while the *Rhizopus sp.* enzyme maintained 100% activity at pH 5 and over 70% at pHs 4, 6, and 7 under the same conditions.^28^

After 6 h of incubation, the endoglucanase produced by *Aspergillus oryzae* ATCC 10124 cultivated on rice husk retained around 80% activity in the pH range of 3–5. When the same microorganism was grown on peanut husk, the enzyme displayed broader pH stability, maintaining approximately 80% of its activity after 6 h within the pH range of 3–6.^7^

#### 3.3.3 Effect of Salts and Organic Compounds

Among the various ions tested, Ca^2^^+^ exhibited the most pronounced positive effect on enzymatic activity, enhancing it by over 60% compared to the control (Table 6), followed closely by Co^2^^+^ (+57%). Other metal ions, including Zn^2^^+^, Fe^2^^+^, Mg^2^^+^, Cu^2^^+^, and Na^+^, also demonstrated beneficial effects, likely through the formation of coordination complexes that modulate electron donation or withdrawal.^6^ These interactions can alter the structural conformation of enzymes by binding to amino or carboxyl groups of amino acid residues, which may result in either the inhibition or enhancement of enzymatic activity.^10^

**Table 6:** Effect of Different Salts and Organic Compounds (at a Concentration of 2 mmol/L) on the Activity of Endoglucanase from *A. niger* ATCC 1004

salts and organic compounds	residual activity (%)
sample control	100 ± 1
CaCl_2_	167 ± 2
CoSO_4_	158 ± 2
CaCO_3_	146 ± 1
ZnSO_4_	131 ± 1
FeSO_4_	125 ± 2
MgCl_2_	123 ± 0
CuSO_4_	123 ± 0
Zn(C_2_H_3_O_2_)	120 ± 1
FeCl2	116 ± 0
Na_2_CO_3_	110 ± 1
Na_2_SO_4_	101 ± 2
Al (NO_3_)_3_	99 ± 0
Pb(C_2_H_3_O_2_)	96 ± 0
MgSO_4_	88 ± 2
KCl	87 ± 1
Triton X-100	46 ± 0
EDTA	12 ± 2
Trolox	10 ± 1

Previous studies have reported increased catalytic activity of endoglucanase in the presence of Mg^2^^+^ and Cu^2^^+^ ions,^43^ whereas decreased activity was associated with Pb^2^^+^, Pb^+^, and K^+^ ions,^43^ but a reduction in activity was observed with Pb^2+^, Pb^+^, and K^+^ ions.^11^ It is well documented that ions and heavy metals can oxidize functional groups located on the side chains of amino acids, thereby diminishing enzymatic activity in certain enzymes.^43^

Conversely, compounds such as EDTA, Triton X-100, and Trolox exhibited inhibitory effects on endoglucanase activity. EDTA, a potent chelating agent, reinforces the hypothesis of the metalloenzymatic nature of the enzymatic complex. Inhibitory effects of EDTA and Triton X-100 were also observed in the endoglucanase from *Penicillium roqueforti* ATCC 10110.^43^ Interestingly, Triton X-100 has been shown to enhance β-sheet formation in endoglucanases, which can, paradoxically, increase enzymatic activity under certain conditions.^44^

Cellulases make up the second most widely utilized class of enzymes across multiple industrial sectors. Their global demand continues to rise exponentially, driven by their broad applicability in processes such as bioethanol production, lactic acid fermentation, biohydrogen generation, detergent formulation, paper manufacturing, and cellulose bioconversion.^30^ A thorough understanding of how salts and organic compounds influence their activity is crucial for optimizing their industrial applications, enabling more robust and efficient bioprocesses.

#### 3.3.4 Effect of Solvents

The logarithm of the partition coefficient (Log *P*) serves as an indicator of a substance’s hydrophobicity and its affinity for organic solvents. Solvents with Log *P* values below 1.0 are frequently classified as hydrophilic, while those above 4.0 are considered hydrophobic. Higher Log *P* values tend to indicate greater hydrophobicity and affinity for organic phases.^45^ Solvent polarity can significantly influence enzyme behavior, potentially causing inactivation or activation by altering the enzyme’s surrounding microenvironment and, consequently, stabilizing conformations favorable or unfavorable for catalysis.^43^

The microenvironment surrounding the enzyme contributes to stabilizing conformations favorable for catalysis. In this study, solvents with Log *P* values ranging from −1.35 to 3.98 were evaluated. The results demonstrated that DMSO, methanol, diethyl ether, and dichloromethane enhanced the catalytic activity of endoglucanase (Table 7). In contrast, acetone, a moderately polar solvent (Log *P*: −0.24), slightly reduced enzymatic activity, maintaining over 90% of the initial activity during 15 min reactions at 20 and 30% (v/v) concentrations. In contrast, highly polar solvents such as DMF and acetonitrile, characterized by low Log *P* values, caused a decrease in the enzymatic activity with increasing concentrations. This reduction is likely due to the disruption of the enzyme’s essential hydration layer, which is critical for maintaining its catalytic functionality.^45^

**Table 7:** Impact of Different Concentrations of Organic Solvents on the Activity of Endoglucanase from *A. niger* ATCC 1004, Results Expressed in Relative Activity (%) where the Enzymatic Activity of the Sample without the Addition of the Organic Solvent Was Regarded as the Control (100%)^a^

		relative activity (% solvent)		
solvent	log *P*	0	20	30
**dimethyl sulfoxide (DMSO)**	–1.35	100	170 ± 1	151 ± 1
**dichloromethane**	1.5	100	132 ± 4	111 ± 0
**ethyl ether**	0.89	100	106 ± 1	134 ± 6
**methanol**	–0.77	100	99 ± 0	146 ± 0
**acetone**	–0.24	100	93 ± 3	94 ± 0
**hexane**	3.98	100	61 ± 2	68 ± 0
**dimethylformamide (DMF)**	–1.01	100	39 ± 0	36 ± 2
**acetonitrile**	–0.45	100	33 ± 0	24 ± 0

a Legend: the Log *P* is the logarithm of the partition coefficient, *P*, of the solvent between *n*-octanol and water and is used as a quantitative measure of the solvent’s polarity.

The partition coefficient is a key parameter for understanding how solvent polarity affects the structure–activity relationship of enzymes. Defined as the logarithm of the solvent’s partition coefficient in an octanol/water system, it reflects the solvent’s hydrophobicity. Hydrophobic solvents, due to their low affinity for water, are generally unable to displace the hydration shell surrounding the enzyme, thus preserving its native conformation and catalytic functionality. In contrast, significant water loss in aqueous environments, often induced by hydrophilic solventes, can lead to enzyme deactivation.^45^

The addition of 20–30% (v/v) ethyl ether (Log *P*: 0.89) or dichloromethane (Log *P*: 1.5), both with relatively high Log *P* values, led to increased enzymatic activity. These solvents likely preserved the hydration layer critical to enzymatic stability and activity.^24^ Overall, hydrophilic solvents were more inhibitory to endoglucanase activity than hydrophobic ones. However, the enzyme demonstrated notable resilience, maintaining or even enhancing its activity in the presence of both solvent types. Remarkably, higher concentrations of methanol and DMSO stimulated hydrolytic activity, while hexane, despite its hydrophobic nature, reduced activity, although approximately 60% of the initial catalytic function was retained.

The enzyme demonstrated remarkable tolerance to solvents such as DMSO, methanol, ethyl ether, acetone, and dichloromethane, exhibiting both activation and stability in aqueous–organic mixtures. This behavior suggests its ability to resist denaturation by organic solvents, likely due to the formation of multiple hydrogen bonds with water molecules. Such interactions confer structural flexibility and conformational adaptability, essential for maintaining efficient catalytic performance under solvent-rich conditions.^46^ Therefore, the studied endoglucanase presents notable solvent stability, reinforcing its potential for applications in organic synthesis.

These findings are consistent with previous reports.^43^ For instance, a study on endoglucanase from *Haloarcula* sp. G10 showed increased enzymatic activity and stability in the presence of nonpolar, hydrophobic solvents with higher Log *P* values. In contrast, a marked reduction in activity was observed following exposure to polar organic solvents (Log *P* ≤ −0.3).^47^

#### 3.3.5 Effect of NaCl Addition

The endoglucanase derived from *A. niger* ATCC 1004 demonstrated a robust catalytic performance across a wide range of saline concentrations. Relative activity values reached 260, 297, 317, 322, 345, 378, 318, and 222% at NaCl concentrations of 0.01, 0.05, 1, 2, 3, 4, 5, and 6 mol·L^–^^1^, respectively. The enzyme’s highest activity was observed at 4 mol·L^–^^1^ NaCl, supporting its classification as a halophilic biocatalyst.^6^

This sustained activity under elevated salinity conditions suggests a structural profile marked by a more acidic surface and a reduced hydrophobic core.^44^ While halotolerance is typically a trait associated with bacterial enzymes, its manifestation in this fungal endoglucanase underscores its exceptional biotechnological relevance and warrants further molecular investigation.^6^ By contrast, the endoglucanase from *Botrytis ricini* URM 5627 exhibited a peak activity increase of 75% at 2 mol·L^–^^1^ NaCl, demonstrating lower halotolerance.^17^

The halophilic behavior of enzymes is generally attributed to a constellation of molecular features. Chief among these is the enrichment of acidic residues on the protein surface, which facilitates the formation of a protective hydration shell via ionic interactions with water molecules and metal ions. This shell stabilizes the enzyme in hypertonic environments. Additionally, stabilization may be reinforced by secondary structures (e.g., α-helices and β-sheets) and salt bridge formation between specific amino acids. Together, these structural adaptations enable halotolerant enzymes to remain catalytically competent under extreme salinity, an essential feature for industrial applications involving saline or hypersaline systems.^33^

Acidic or alkaline pretreatment, commonly applied in enzymatic hydrolysis, increases the NaCl concentration in the cellulose substrate. Without a desalination step, the subsequent hydrolysis process must occur under high-salinity conditions, which compromises its efficiency. The use of halotolerant cellulases represents a promising strategy to mitigate, or even eliminate, these negative effects, enabling more efficient and cost-effective biofuel production from lignocellulosic biomass.^33^

### 3.4 Enzymatic Saccharification

Using the enzymatic blend produced by *A. niger* ATCC 1004, rice husk saccharification was carried out, yielding a maximum concentration of reducing sugars of 13.03 ± 0.02 mg/g of RH after 7 h (Figure 3a). Rice husk is rich in carbohydrate polymers such as cellulose and hemicellulose within its cell walls, making it an attractive substrate for bioprocesses such as alcoholic fermentation. However, the lignin present in the lignocellulosic matrix acts as a physical and chemical barrier, significantly hindering enzymatic hydrolysis.^48^

**Figure 3:**
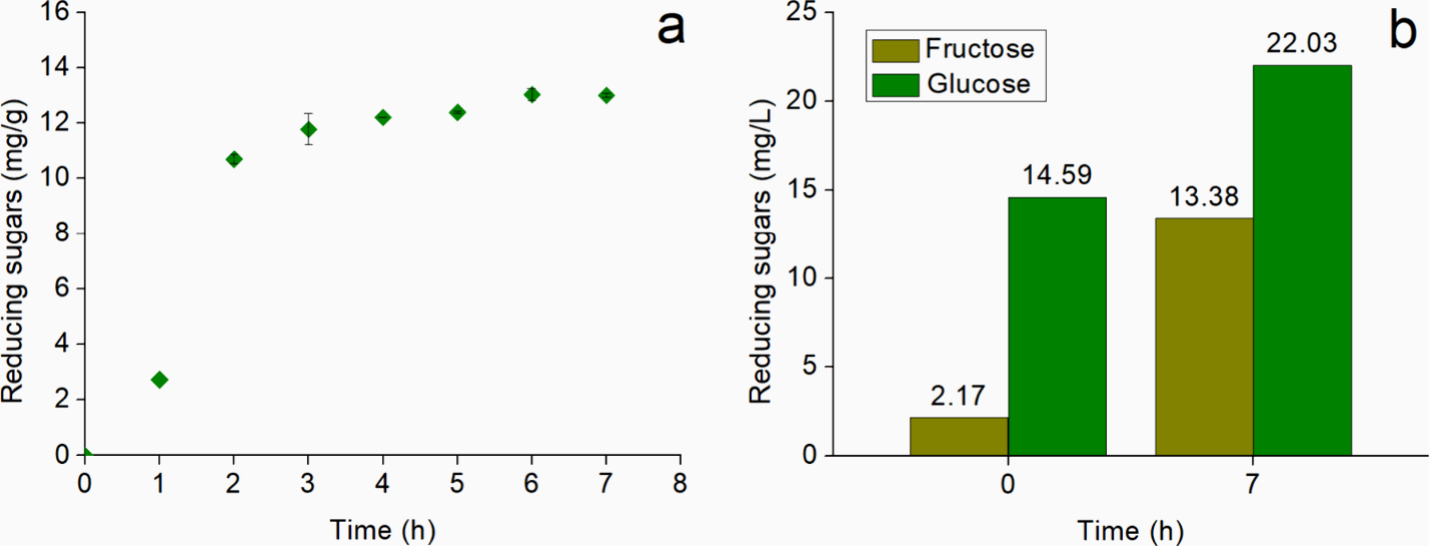
(a) Saccharification of rice husk using enzymatic blend from *A. niger* ATCC 1004; (b) individual sugar composition before and after enzymatic hydrolysis, determined by HPLC.

Typically, pretreatment steps are required to remove this barrier and expose the carbohydrates to an enzymatic action. In contrast, this study demonstrates effective enzymatic hydrolysis without the need for chemical or physicochemical pretreatment; only drying and grinding of the biomass were performed. This upstream strategy highlights the potential for reducing operational costs in biotechnological processes. Similarly, other studies also report the preparation of lignocellulosic biomass, such as rice husk, through air drying and grinding for efficient bioconversion and bioethanol production, highlighting the potential of minimal pretreatment strategies for sustainability and economic purposes.^49^

Furthermore, the use of an enzymatic blend, containing endoglucanase (0.435 U/mL) and other synergistic hydrolytic enzymes, is advantageous when compared to purified enzymes. This enzymatic consortium enhances lignocellulosic degradation efficiency.^50^ Enzymatic saccharification also offers clear benefits over chemical hydrolysis, such as higher specificity, reduced energy demand, and lower overall processing costs.^17^ Notably, these features have driven growing interest in enzymatic saccharification, especially in bioethanol production within the current biotechnological landscape.^50^

The HPLC analysis revealed the release of 7.24 mg/L glucose and 11.21 mg/L fructose monosaccharides (Figure 3b). The saccharification of rice husks using an enzymatic blend, without prior pretreatment other than drying and grinding, resulted in a remarkable 51% increase in glucose concentration and an impressive 513% increase in fructose levels. Notably, the final fructose-to-glucose ratio exceeded values reported in the literature, which generally indicate a predominance of glucose.^18^ This process yielded a fructose content 50% higher than that obtained by conventional methods. Since rice husks do not naturally contain fructose in their polysaccharide composition, the presence of glucose isomerase in the enzymatic blend likely explains this outcome by catalyzing the conversion of d-glucose into d-fructose.^51^

The observed isomerization from aldose to ketose is attributed to the complex enzymatic composition of the blend. The d-glucose released by the enzymatic blend is subsequently converted into d-fructose, increasing its yield and enabling the production of a high-fructose syrup. This noncommercial enzymatic mixture offers a broad spectrum of catalytic activities, including xylanase, exoglucanase, and isomerases, contributing to the efficiency and versatility of the saccharification process.^50^

Traditionally, producing a fructose-rich syrup from food waste requires separate saccharification and isomerization steps, a method marked by low efficiency and potential dependency on metal cofactors and other additives.^51^ This study introduces a streamlined one-step process using an enzymatic blend, eliminating the need for pretreatment or postsaccharification enzyme supplementation.

High-fructose corn syrup (HFCS) is a widely used sweetener and ranks as the second most consumed sugar source globally, underscoring the urgent need to diversify production routes for this critical ingredient.^51^ With approximately twice the sweetness of sucrose at equal caloric value, HFCS plays an essential role in the manufacture of low-glycemic index products, holding strategic importance in the food and beverage industries.^52^ The proposed process offers a novel biotechnological approach for generating fructose-rich syrup from low-cost rice husks, providing compelling advantages in terms of cost, efficiency, and sustainability.

The high-fructose syrup developed from rice husk presents a viable alternative to HFCS and can also serve as a substrate for second-generation ethanol production using yeast strains such as *Saccharomyces cerevisiae*, butanol via *Clostridium* species, or as input in bioprocesses for synthesizing other high-value compounds such as xanthan gum, rhamnolipids, and rare sugars such as fucose, thus expanding the technological potential of this platform.

## 4 Conclusions

The present study demonstrated the biotechnological potential of an enzymatic blend produced by *A. niger* ATCC 1004 via SSF, using low-cost lignocellulosic biomass (cocoa bean shell, forage palm, and rice husk). Process optimization, based on endoglucanase activity, indicated the ideal conditions as 80% cocoa bean shell, 20% forage palm, 28 °C, 60% moisture, and 96 h.

The endoglucanase exhibited remarkable thermal and operational stabilities, with high activity between pHs 5 and 9, and tolerance to temperatures up to 70 °C. It also stood out for its tolerance in extreme saline conditions (the maximum activity with 4 mol·L^–^^1^ NaCl), activation by metal ions (Ca^2^^+^ and Co^2^^+^), and robustness in the presence of organic solvents such as DMSO (+69.56%), indicating strong potential for industrial applications.

The enzymatic extract enabled the direct saccharification of rice husk, without the need for physicochemical or biological pretreatments, releasing 13 mg/g of the reducing sugars. HPLC analysis confirmed the release of glucose and fructose (11.21 mg/L), suggesting the presence and synergistic action of glucose isomerase (or enzymes with a similar function) in the crude extract.

This approach represents an innovative and sustainable biotechnological route for the production of fructose-rich syrups and the valorization of food waste, contributing viable solutions for biorefineries and the advancement of the circular bioeconomy.

## Data Availability

Data will be made available on request.
